# Walking Speed and Risk of Cancer in Two Prospective Cohort Studies

**DOI:** 10.1002/jcsm.13792

**Published:** 2025-04-24

**Authors:** Jonathan K. L. Mak, Kathryn Choon Beng Tan, Juulia Jylhävä, Sara Hägg, Ching‐Lung Cheung

**Affiliations:** ^1^ Department of Pharmacology and Pharmacy, Li Ka Shing Faculty of Medicine The University of Hong Kong Hong Kong SAR China; ^2^ Department of Medical Epidemiology and Biostatistics, Karolinska Institutet Stockholm Sweden; ^3^ Department of Medicine, School of Clinical Medicine, Li Ka Shing Faculty of Medicine The University of Hong Kong Hong Kong SAR China; ^4^ Faculty of Medicine and Health Technology and Gerontology Research Center (GEREC) Tampere University Tampere Finland; ^5^ Tampere Institute for Advanced Study Tampere Finland; ^6^ Hinda and Arthur Marcus Institute for Aging Research, Hebrew SeniorLife Boston Massachusetts USA; ^7^ Laboratory of Data Discovery for Health (D24H), Hong Kong Science Park, Pak Shek Kok Hong Kong SAR China

**Keywords:** cancer, cohort study, gait speed, physical activity, sarcopenia

## Abstract

**Background:**

Walking speed is a reliable marker of sarcopenia and a strong predictor of mortality, but its relationship with cancer incidence remains largely unexplored. We aimed to investigate the association between walking speed and the risk of any cancer and five common cancers, including lung, breast, colorectum, prostate, and stomach, and to explore potential mediation by biomarkers of inflammation, and lipid and glucose metabolism.

**Methods:**

The primary analysis was conducted in 431 598 participants from the UK Biobank (mean age 56.3 [SD 8.1] years at baseline), and the generalizability of findings was further tested in 1311 participants from the Hong Kong Osteoporosis Study (HKOS; mean age 57.8 [SD 11.9] years). Walking speed was self‐reported in the UK Biobank and measured using a timed 6‐m walk test in the HKOS. Incident cancer cases were identified from electronic health records. We used Cox models, adjusted for age, sex, height, body mass index, socioeconomic, lifestyle factors, family history of cancer, and grip strength, to estimate the association between walking speed and cancer incidence. Single and multiple mediator models were performed in the UK Biobank to examine the mediating effects of C‐reactive protein (CRP), white blood cell (WBC) count, total cholesterol, low‐density lipoprotein (LDL) cholesterol, and glucose levels.

**Results:**

Over a median follow‐up of 10.9 and 6.9 years, 11.7% and 5.0% of the UK Biobank and HKOS participants were diagnosed with cancer, respectively. In the UK Biobank, those reported a brisk vs. slow walking pace had a 13% lower risk of any cancer (95% CI 0.84–0.90). Similarly, HKOS participants with a faster walking speed (≥ 1.0 vs. < 1.0 m/s) had a 45% reduced risk of any cancer (95% CI 0.31–0.98). In the UK Biobank, brisk walking pace was associated with a significantly decreased risk of lung cancer (hazard ratio [HR] 0.47, 95% CI 0.42–0.53) and a slightly increased risk of prostate cancer (HR 1.11, 95% CI 1.02–1.21). CRP, WBC count, total cholesterol, and LDL cholesterol significantly mediated the association between brisk walking pace and any cancer, with proportions of mediation being 6.4% (95% CI 4.4–8.7%), 11.4% (8.4–17.1%), 9.3% (7.1–12.9%), and 8.3% (6.1–11.9%), respectively. The combined mediated proportion of all five potential mediators was 25.9% (19.5–37.2%).

**Conclusion:**

Faster walking speed, whether self‐reported or measured, is associated with a reduced risk of cancer development. This association appears to be partially mediated by lower inflammation and improved lipid profiles.

## 1 Introduction

Cancer remains a significant public health concern, ranking among the leading causes of death [1]. In 2022, there were nearly 20 million new cancer cases and 9.7 million cancer deaths worldwide [1], highlighting the need for effective cancer prevention strategies. Physical activity is a well‐established protective factor against various cancers, including lung, colon, breast, and liver cancer [2]. Current guidelines recommend engaging in regular moderate‐to‐vigorous physical activity, in combination with muscle‐strengthening activity, for cancer prevention [3]. Walking, as the most common and accessible form of moderate physical activity, has been a key focus in promoting physical activity [4]. Recent research has also demonstrated an inverse relationship between daily step count and step intensity with the risk of cancer incidence and mortality [5].

Beyond the amount of walking, the pace at which one walks correlates with higher relative activity intensity, which may produce greater physiological response and confer additional health benefits [5, 6]. Walking speed is a quick, sensitive, objective, and highly reliable marker of functional status and overall health, especially in older adults [7]. Since gait speed could reflect the health status of multiple body systems, it has been shown to be a strong predictor of age‐related diseases including cardiovascular diseases [8] and dementia [9], as well as mortality [6]. Moreover, walking speed is an important measure for assessing sarcopenia [10] and physical frailty in clinical practice [11]. As skeletal muscle plays a crucial role in regulating inflammatory and metabolic pathways, sarcopenia is linked to chronic inflammation and dysfunction of lipid and glucose metabolism [12], which may contribute to cancer progression [13, 14].

However, whether walking speed per se is associated with cancer incidence remains poorly studied. In a previous observational study, Kwan et al. found no significant association between walking speed and invasive breast cancer incidence among 14 719 postmenopausal women in the Women's Health Initiative study [15]. Conversely, emerging evidence from Mendelian randomization studies indicates a potential genetic causal relationship between a faster walking pace and a lower risk of breast cancer [16] and lung cancer [17], suggesting that gait speed may not simply be a marker, but also a potential contributor to cancer development. Nevertheless, given the limited evidence and potential limitations and biases inherent in Mendelian randomization, such as limited statistical power as genetic instruments can only explain a small variance, and the presence of weak instrument bias and horizontal pleiotropy [16, 17], more studies with larger sample sizes and longer follow‐up periods are needed to elucidate the association between walking speed and the risk of various cancers, and to explore potential underlying mechanisms.

The aim of the present study was to investigate the relationship between walking speed and the incidence of any cancer and the five most frequently diagnosed cancers worldwide, including lung, female breast, colorectal, prostate, and stomach cancers [1]. We hypothesized that a faster walking speed would be associated with a lower risk of developing these cancers. Additionally, we performed mediation analyses to assess the extent to which these associations could be mediated by biomarkers of inflammation, and lipid and glucose metabolism.

## 2 Methods

### 2.1 Study Population

This is a prospective cohort study using data from two sources, including the UK Biobank (as a primary cohort) and the Hong Kong Osteoporosis Study (HKOS; as a validation cohort). The UK Biobank is a population‐based cohort which has recruited over 500 000 individuals aged 37–73 from England, Wales, and Scotland between 2006 and 2010 [18]. During baseline assessment, participants underwent extensive testing, including touch‐screen questionnaires, physical measurements, and collection of biological samples [18]. The UK Biobank participants were followed through linkage to electronic medical records, including the national death and cancer registries. The HKOS is the first longitudinal cohort study on osteoporosis and fracture of all ages in Asia, which has enrolled over 9000 community‐dwelling Chinese adults from 1995 to 2010 [19]. Between 2015 and 2019, a full‐scale follow‐up study was conducted, consisting of 1386 participants aged 27–96 years who completed questionnaires, clinical assessments (such as gait speed and grip strength), and provided biomedical data [19]. The HKOS participants are linked to the Clinical Data Analysis and Reporting System (CDARS), the territory‐wide electronic health record database managed by the Hospital Authority of Hong Kong, which captures both inpatient and outpatient records and covers >80% of all hospital admissions in Hong Kong.

This study is part of the UK Biobank registered project 22224. Ethical approval for the UK Biobank study was obtained from the North West Multi‐centre Research Ethics Committee (approval number: 11/NW/0382), and this analysis was also approved by the Regional Ethics Board in Stockholm (Dnr 2016/1888–31/1). The HKOS study was approved by the Institutional Review Board of the University of Hong Kong/HA HKW, HKSAR, China (reference numbers UW 15‐236 and UW 23‐396). All participants provided informed consent prior to data collection.

For this analysis, we excluded individuals who had withdrawn from the study, had a cancer diagnosis before baseline, or had missing data on walking speed and covariates (Figure S1). This resulted in a final sample size of *n* = 431 598 participants from the UK Biobank and *n* = 1311 participants from the HKOS.

### 2.2 Walking Speed

In the UK Biobank, walking speed was assessed using a self‐reported question: “How would you describe your usual walking pace?”. Participants were provided with three options of “slow”, “steady/average”, or “brisk”, which correspond to walking speeds of < 3 miles per hour (approximately 1.34 m/s), 3–4 miles per hour (approximately 1.34–1.79 m/s), and > 4 miles per hour (approximately 1.79 m/s), respectively. Those who answered “None of the above” or “Prefer not to answer” were excluded from the analysis.

In the HKOS, a timed 6‐m walk test was performed during the in‐person assessments between 2015 and 2019. Participants were instructed to walk along a straight line of 6 m long at a comfortable pace. Walking speed (in m/s) was then calculated by dividing the distance (6 m) by the time walked. Following recommendations of the Asian Working Group for Sarcopenia [20], we categorized walking speed into ≥ 1.0 and < 1.0 m/s, where a 6‐m walking speed of < 1.0 m/s is typically considered indicative of low physical performance [20].

### 2.3 Outcomes

Cancer cases in the UK Biobank were ascertained from the cancer registries in England, Wales, and Scotland, with complete follow‐up available through February 29, 2020. In the HKOS, cancer cases were identified from the electronic health records in CDARS, with follow‐up available until January 15, 2024. We defined cancer of all sites (excluding non‐melanoma skin cancer) and five specific cancers (lung, female breast, colorectum, prostate, and stomach) using the International Classification of Diseases (ICD) 9th or 10th revision codes, as presented in Table S1. Participants were followed from the date of baseline assessment until the first occurrence of the corresponding cancer diagnosis, date of death, or end of follow‐up, whichever came first.

### 2.4 Covariates

Information on demographics, socioeconomic indicators, lifestyle factors, family history of cancer, and grip strength was collected during baseline assessment. Body mass index (BMI) was calculated using measured weight and height for both cohorts. Ethnicity was self‐reported and categorized as white, Asian, black, or other in the UK Biobank; all participants from the HKOS were Asians. Education was determined by the highest self‐reported qualification and categorized as primary or below, secondary, or college/university degree. Socioeconomic deprivation was assessed using the Townsend deprivation index in the UK Biobank, which was derived from national census data; this information is not available in the HKOS. Smoking status was categorized as never, previous, or current smoker. Alcohol consumption was categorized as never, occasionally, once a month, or once a week. Physical activity was assessed using the International Physical Activity Questionnaire in the UK Biobank [21] and a physical activity questionnaire developed for Hong Kong Chinese adults in the HKOS [22]. In both cohorts, the total metabolic equivalent of task (MET) in minutes per week was calculated based on the weekly frequency and duration of vigorous, moderate, and walking activity [23]. Family history of cancer in father, mother, or siblings was self‐reported in both cohorts. Grip strength was measured using a Jamar hydraulic hand dynamometer in both cohorts. We took the mean of the right and left hand values in the UK Biobank [24] and the mean of the three repeated measurements of the dominant hand in the HKOS for the analysis [25].

### 2.5 Potential Mediators

We considered two biomarkers of inflammation (C‐reactive protein [CRP], white blood cell [WBC] count) [13] and three biomarkers of lipid and glucose metabolism (total cholesterol, low‐density lipoprotein [LDL] cholesterol, glucose) [14] as potential mediators. Blood samples were collected at the UK Biobank baseline assessment. Serum CRP (mg/L), total cholesterol (mmol/L), LDL cholesterol (mmol/L), and glucose levels (mmol/L) were measured on the Beckman Coulter AU5800 chemistry analyser (https://biobank.ndph.ox.ac.uk/ukb/ukb/docs/serum_biochemistry.pdf). CRP was measured by the immunoturbidimetric method; total cholesterol was measured by cholesterol oxidase‐peroxidase (CHO‐POD) analysis; LDL cholesterol was measured by enzymatic protective selection analysis; and glucose was measured by hexokinase analysis. WBC count in whole blood (×10^9^ cells/L) was measured using the Coulter LH 750 System (https://biobank.ndph.ox.ac.uk/ukb/ukb/docs/haematology.pdf). All the biomarkers were considered as continuous variables and were standardized to mean = 0 and standard deviation (SD) = 1. Mediation analyses were performed only in the UK Biobank as the inflammation biomarkers were not available in the HKOS.

### 2.6 Statistical Analysis

Sample characteristics were summarized as means and SDs for continuous variables and proportions for categorical variables. Univariate comparisons between exposure groups (walking speed) and outcome groups (cancer) were performed using independent *t* tests, analysis of variance, or *χ* ^2^ tests as appropriate. Kaplan–Meier curves were plotted to examine the probability of any cancer incidence by walking speed categories in the UK Biobank and HKOS.

We fitted Cox proportional‐hazards models in the UK Biobank to estimate hazard ratios (HR) with 95% confidence intervals (CI) for the risk of developing any cancer and site‐specific cancers. The models were first adjusted for age and sex (model 1) and were further adjusted for height, BMI, baseline assessment centre, ethnicity, education, Townsend deprivation index, smoking, alcohol consumption, physical activity, and family history of cancer (model 2). Additionally, we adjusted the models for grip strength (model 3), which is a related marker of sarcopenia and frailty that has also been linked to cancer risk [24]. For the cancers that were significantly associated with walking speed, subgroup analyses were performed to test if the associations may differ by age at baseline (< 65 vs. ≥ 65 years), sex (women vs. men), ethnicity (white vs. non‐white), smoking status (never‐smokers vs. ever‐smokers) BMI (< 25 vs. ≥ 25 kg/m^2^), and physical activity level (low vs. moderate/high).

For validation of the results in the HKOS, we fitted Cox models to examine the association between measured walking speed and risk of any cancer. The models were adjusted for same covariates as in the UK Biobank, except baseline assessment centre (since all HKOS participants attended the same assessment centre) and socioeconomic deprivation (which was not available in the HKOS). In addition, a restricted cubic spline model was used to assess the dose–response relationship between timed walking speed and risk of any cancer in the HKOS. Subgroup analyses were performed in the HKOS by age at baseline (< 65 vs. ≥ 65 years) and sex (women vs. men).

To explore the mediating roles of CRP, WBC count, total cholesterol, LDL cholesterol, and glucose in the association between walking speed and cancer, we conducted mediation analyses in the UK Biobank using the “CMAverse” R package [26]. The regression‐based approach was used to estimate the total effect, pure natural direct effect (PNDE), and total natural indirect effect (TNIE), under the strong assumption that there is no unmeasured confounding [26]. PNDE represents the effect of walking speed on cancer that is independent of the mediator(s), while TNIE represents the effect of walking speed on cancer that is mediated by the mediator(s). Total effect corresponds to the sum of PNDE and TNIE, and proportion mediated (i.e. magnitude of mediation) was calculated as TNIE divided by the total effect. Of note, while the term “effect” is commonly used in mediation analyses, it does not directly imply causality in our findings. We performed both single mediator and parallel multiple mediator models for any cancer and lung cancer, which aimed to quantify the proportion of mediation through each mediator and all five mediators combined, respectively. All mediation models were adjusted for age, sex, height, BMI, baseline assessment centre, ethnicity, education, Townsend deprivation index, smoking, alcohol consumption, physical activity, family history of cancer, and grip strength. Effect estimates were presented as HRs, and 95% CIs were obtained using bootstrapping with 200 repetitions.

We performed two sensitivity analyses. Firstly, as we observed an unexpected positive association between walking speed and prostate cancer in the UK Biobank, we further considered serum testosterone level [27] and diabetes [28] as potential confounders in the models. Secondly, to minimize the possibility of reverse causality, we repeated the analysis between walking speed and any cancer and the significant cancer types in the UK Biobank and HKOS by excluding the first 3 years of follow‐up.

We considered a *p* < 0.05 as nominal significance. To account for multiple testing in the UK Biobank, we further considered a Bonferroni‐corrected significance threshold of *p* < 0.01 (i.e. 0.05/5, accounting for five specific cancer sites examined in the UK Biobank). The proportional‐hazards assumption of the Cox models was verified using Schoenfeld residuals. All analyses were performed in R version 4.3.2.

## 3 Results

### 3.1 Participant Characteristics

The analysis included 431 598 UK Biobank and 1311 HKOS participants, the mean age of which were 56.3 (SD = 8.1) and 57.8 (SD = 11.9) years, respectively (Table 1). The proportion of women was higher in the HKOS (78.9%) compared to the UK Biobank (53.1%). Besides, higher proportions of participants in the HKOS were never‐smokers (94.2% vs. 55.2%), never‐drinkers (65.7% vs. 4.2%), and had a lower mean BMI (23.3 vs. 27.4 kg/m^2^) compared to the UK Biobank. Education and physical activity levels were similar between the two cohorts (Table 1).

**TABLE 1:** Baseline characteristics of the included participants.

Characteristic	UK Biobank (*n* = 431 598)	HKOS (*n* = 1311)
Age, year, mean (SD)	56.3 (8.1)	57.8 (11.9)
Sex, *n* (%)		
Women	229 355 (53.1)	1035 (78.9)
Men	202 243 (46.9)	276 (21.1)
Height, m, mean (SD)	1.69 (0.09)	1.58 (0.08)
BMI, kg/m^2^, mean (SD)	27.4 (4.7)	23.3 (3.7)
Ethnicity, *n* (%)		
White	409 246 (94.8)	—
Asian	9370 (2.2)	1311 (100)
Black	6678 (1.5)	—
Others	6304 (1.5)	—
Education, *n* (%)		
Primary or below	69 255 (16.0)	227 (17.3)
Secondary	216 648 (50.2)	674 (51.4)
College or university	145 695 (33.8)	410 (31.3)
Deprivation index, mean (SD)	−1.36 (3.05)	—
Smoking status, *n* (%)		
Never	238 445 (55.2)	1235 (94.2)
Previous	148 685 (34.4)	43 (3.3)
Current	44 468 (10.3)	33 (2.5)
Alcohol consumption, *n* (%)		
Never	17 905 (4.1)	861 (65.7)
Occasionally	62 457 (14.5)	168 (12.8)
Once a month	47 996 (11.1)	142 (10.8)
Once a week	303 240 (70.3)	140 (10.7)
Physical activity, MET‐min/week, mean (SD)	2789.2 (3827.3)	2737.3 (2965.7)
Family history of cancer, *n* (%)	149 827 (34.7)	342 (26.1)
Grip strength, kg, mean (SD)	31.0 (11.0)	23.9 (7.9)
Any incident cancer, *n* (%)	50 480 (11.7)	66 (5.0)
Died during follow‐up, *n* (%)	21 408 (5.0)	25 (1.9)

Abbreviation: BMI, body mass index; HKOS, Hong Kong Osteoporosis Study; MET, metabolic equivalent of task.

In the UK Biobank, 7.5%, 52.4%, and 40.1% of participants reported a slow, average, and brisk walking pace, respectively, whereas in the HKOS, 16.0% of participants were classified as slow walkers (< 1.0 m/s). Participants with a slower walking speed were generally older, shorter, had higher BMI, lower education, lower physical activity level, and lower grip strength in both cohorts (*p* < 0.05) (Table S2). Moreover, those who developed cancer during the follow‐up period tended to be older, had higher BMI, lower education, and were more likely to be smokers (Table S3).

### 3.2 Walking Speed and Risk of cancer

**FIGURE 1:**
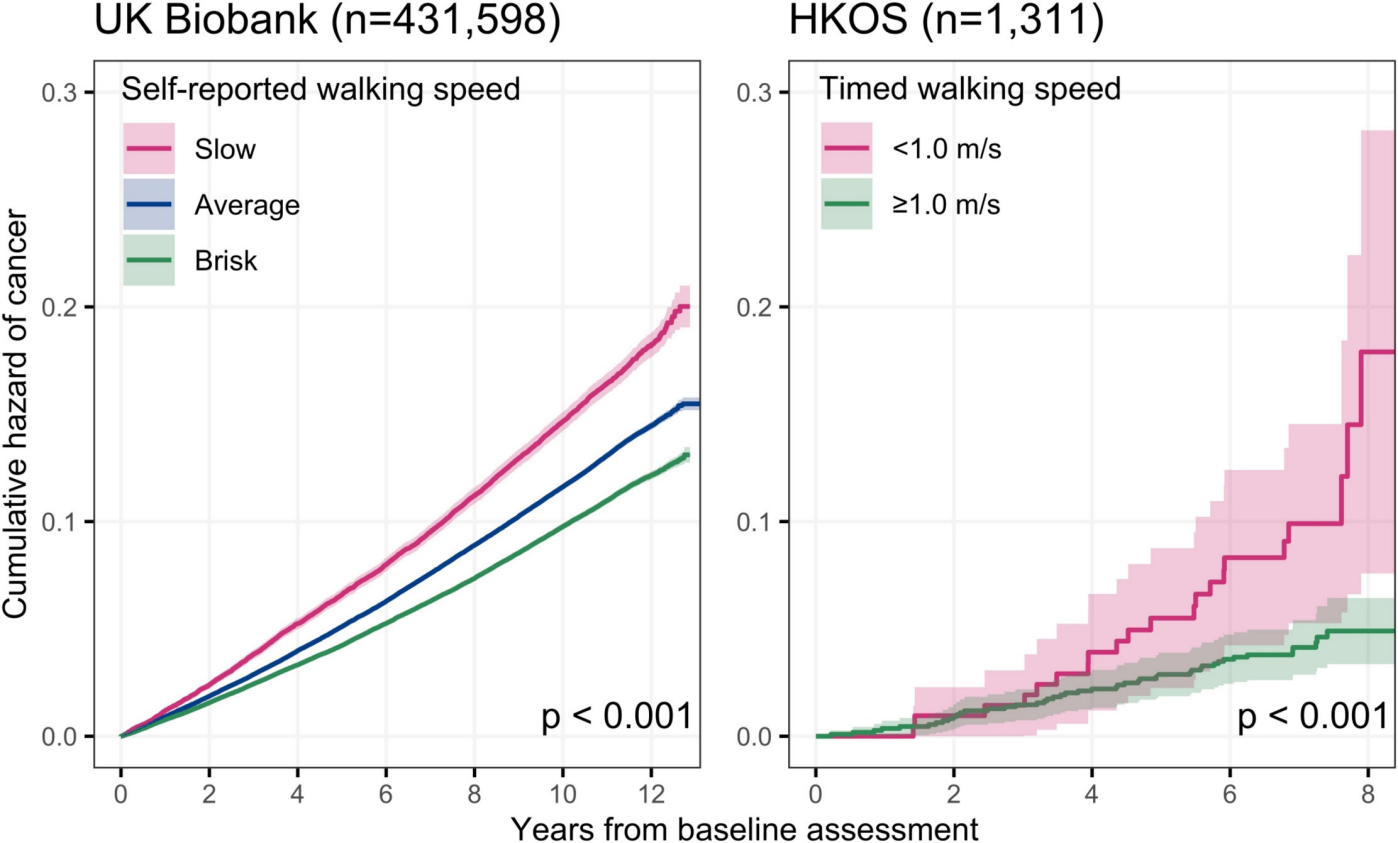
Kaplan–Meier curves of cancer incidence stratified by walking speed in the UK Biobank and the Hong Kong Osteoporosis Study (HKOS). *p* Values were based on log‐rank tests.

During a median follow‐up of 10.9 years (interquartile range [IQR] = 10.0–11.6) in the UK Biobank and 6.9 years (IQR = 6.6–7.8) in the HKOS, 11.7% and 5.0% of participants were diagnosed with incident cancer, respectively (Table 1). Kaplan–Meier curves in Figure 1 show that individuals with a faster walking speed had a lower probability of developing any cancer in both cohorts (log‐rank *p* < 0.001). The incidence rate of any cancer among slow walkers was comparable in the UK Biobank (1485.8 per 100 000 person‐years) and the HKOS (1483.4 per 100 000 person‐years) and was higher than those with a faster walking speed (1181.5 for average walking pace and 993.7 for brisk walking pace in the UK Biobank) (Tables 2 and 3).

**TABLE 2:** Association between self‐reported walking speed and risk of any cancer and site‐specific cancers in the UK Biobank.

Cancer site	Slow	Average	Brisk	*p*_trend_ ^a^
**Any cancer**				
Incidence per 100 000 person‐years	1485.8	1181.5	993.7	—
Model 1, HR (95% CI)^b^	1 (ref)	0.86 (0.84, 0.89)**	0.80 (0.78, 0.83)**	< 0.001
Model 2, HR (95% CI)^c^	1 (ref)	0.91 (0.88, 0.94)**	0.87 (0.84, 0.90)**	< 0.001
Model 3, HR (95% CI)^d^	1 (ref)	0.91 (0.88, 0.94)**	0.87 (0.84, 0.90)**	< 0.001
**Lung cancer**				
Incidence per 100 000 person‐years	206.3	83.4	46.5	—
Model 1, HR (95% CI)^b^	1 (ref)	0.45 (0.42, 0.50)**	0.30 (0.27, 0.33)**	< 0.001
Model 2, HR (95% CI)^c^	1 (ref)	0.62 (0.57, 0.68)**	0.47 (0.42, 0.52)**	< 0.001
Model 3, HR (95% CI)^d^	1 (ref)	0.63 (0.57, 0.69)**	0.47 (0.42, 0.53)**	< 0.001
**Breast cancer in women**				
Incidence per 100 000 person‐years	369.0	346.8	315.3	—
Model 1, HR (95% CI)^b^	1 (ref)	0.96 (0.89, 1.05)	0.91 (0.84, 0.99)*	0.004
Model 2, HR (95% CI)^c^	1 (ref)	1.00 (0.91, 1.09)	0.95 (0.87, 1.05)	0.12
Model 3, HR (95% CI)^d^	1 (ref)	1.00 (0.91, 1.09)	0.95 (0.87, 1.05)	0.12
**Colorectal cancer**				
Incidence per 100 000 person‐years	147.4	123.5	99.5	—
Model 1, HR (95% CI)^b^	1 (ref)	0.93 (0.84, 1.02)	0.85 (0.77, 0.94)*	< 0.001
Model 2, HR (95% CI)^c^	1 (ref)	0.97 (0.88, 1.08)	0.92 (0.82, 1.03)	0.048
Model 3, HR (95% CI)^d^	1 (ref)	0.98 (0.88, 1.08)	0.92 (0.83, 1.03)	0.06
**Prostate cancer in men**				
Incidence per 100 000 person‐years	487.0	509.7	457.8	—
Model 1, HR (95% CI)^b^	1 (ref)	1.22 (1.12, 1.32)**	1.29 (1.19, 1.40)**	< 0.001
Model 2, HR (95% CI)^c^	1 (ref)	1.11 (1.02, 1.20)*	1.13 (1.03, 1.23)**	0.040
Model 3, HR (95% CI)^d^	1 (ref)	1.10 (1.01, 1.19)*	1.11 (1.02, 1.21)*	0.08
**Stomach cancer**				
Incidence per 100 000 person‐years	28.0	14.3	11.2	—
Model 1, HR (95% CI)^b^	1 (ref)	0.57 (0.45, 0.71)**	0.51 (0.40, 0.66)**	< 0.001
Model 2, HR (95% CI)^c^	1 (ref)	0.81 (0.63, 1.03)	0.87 (0.66, 1.16)	0.69
Model 3, HR (95% CI)^d^	1 (ref)	0.80 (0.62, 1.02)	0.86 (0.65, 1.14)	0.63

Abbreviations: CI, confidence interval; HR, hazard ratio.

*p < 0.05, **p < 0.01.

^a^ The *p* value for linear trend was calculated using walking speed as an ordinal variable (slow = 1, average = 2, brisk = 3).

^b^ Model 1: adjusted for age and sex.

^c^ Model 2: model 1 + height, body mass index, baseline assessment centre, ethnicity, education, deprivation, smoking, alcohol consumption, physical activity, and family history of cancer.

^d^ Model 3: model 2 + grip strength.

**TABLE 3:** Association between timed walking speed and risk of any cancer in the Hong Kong Osteoporosis Study.

Any cancer	< 1.0 m/s	≥ 1.0 m/s
Incidence per 100 000 person‐years	1483.4	587.7
Model 1, HR (95% CI)^a^	1 (ref)	0.56 (0.32, 0.98)*
Model 2, HR (95% CI)^b^	1 (ref)	0.52 (0.29, 0.92)*
Model 3, HR (95% CI)^c^	1 (ref)	0.55 (0.31, 0.98)*

Abbreviations: CI, confidence interval; HR, hazard ratio; SD, standard deviation.

* *p* < 0.05.

^a^ Model 1: adjusted for age and sex.

^b^ Model 2: model 1 + height, body mass index, ethnicity, education, smoking, alcohol consumption, physical activity, and family history of cancer.

^c^ Model 3: model 2 + grip strength.

In the UK Biobank, adjusted for age, sex, height, BMI, baseline assessment centre, ethnicity, education, deprivation, smoking, alcohol consumption, physical activity, and family history of cancer (model 2), there was a significantly reduced risk of any cancer among those with an average (HR = 0.91, 95% CI = 0.88–0.94) or brisk walking pace (HR = 0.87, 95% CI = 0.84–0.90) compared to those with a slow walking pace (*p*‐value for linear trend < 0.001) (Table 2). This association remained unchanged when further adjusted for grip strength (model 3) (Table 2).

The HKOS was used as a validation cohort to examine the association between measured walking speed and the risk of any cancer. Consistent with the results found in the UK Biobank, HKOS participants who walked at a speed of ≥ 1.0 vs. < 1.0 m/s had a lower risk of any cancer (HR = 0.55, 95% CI = 0.31–0.98) (Table 3). The relationship between the continuous measure of walking speed and any cancer in the HKOS is presented in Figure S2, which shows that a slower walking speed was associated with an increased risk of any cancer, but this association was less evident when walking speed exceeded 1.2 m/s.

When stratified by cancer types in the UK Biobank, brisk walking pace was associated with a significantly lower risk of lung cancer (HR = 0.47, 95% CI = 0.42–0.53). On the contrary, brisk walking pace was associated with a slightly higher risk of prostate cancer (HR = 1.11, 95% CI = 1.02–1.21), although this association was not statistically significant after applying the Bonferroni correction. No statistically significant association was found between walking speed and breast, colorectal, and stomach cancers in the fully adjusted models (Table 2).

### 3.3 Mediation Analysis

After adjusting for potential confounders, CRP, WBC count, total cholesterol, and LDL cholesterol showed statistically significant partial mediating effects on the association between walking speed and any cancer in the UK Biobank (Table 4). The proportions of mediation for the association comparing brisk vs. slow walking speed were 6.4% (95% CI = 4.4–8.7%) for CRP, 11.4% (8.4–17.1%) for WBC count, 9.3% (7.1–12.9%) for total cholesterol, and 8.3% (6.1–11.9%) for LDL cholesterol. Glucose did not significantly mediate the walking speed‐cancer association. In the multiple mediator model, the total mediated proportion by the five mediators combined was 25.9% (95% CI = 19.5–37.2%) (Table 4). The association between brisk walking pace and lung cancer was also significantly mediated, albeit to a lesser extent, by CRP, WBC count, total cholesterol, and LDL cholesterol, with proportions of mediation ranged from 2.0 to 2.3% and a combined mediated proportion of 5.9% (4.5–7.9%) (Table S4).

**TABLE 4:** Mediation analysis for the association between self‐reported walking speed and any cancer, for biomarkers of inflammation and lipid metabolism in the UK Biobank.

Mediators	Total effect^a^, HR (95% CI)	Pure natural direct effect^b^, HR (95% CI)	Total natural indirect effect^c^, HR (95% CI)	Proportion mediated^d^, % (95% CI)
Brisk vs. slow				
CRP	0.87 (0.84, 0.90)	0.88 (0.85, 0.91)	0.99 (0.99, 0.99)	6.4 (4.4, 8.7)
WBC count	0.87 (0.84, 0.90)	0.89 (0.86, 0.92)	0.98 (0.98, 0.99)	11.4 (8.4, 17.1)
Total cholesterol	0.87 (0.85, 0.90)	0.88 (0.86, 0.91)	0.99 (0.98, 0.99)	9.3 (7.1, 12.9)
LDL cholesterol	0.87 (0.83, 0.91)	0.88 (0.85, 0.92)	0.99 (0.99, 0.99)	8.3 (6.1, 11.9)
Glucose	0.87 (0.84, 0.91)	0.88 (0.85, 0.91)	1.00 (0.99, 1.00)	0.9 (−0.0, 2.0)
All five	0.87 (0.85, 0.91)	0.91 (0.88, 0.94)	0.97 (0.96, 0.97)	25.9 (19.5, 37.2)
Average vs. slow				
CRP	0.91 (0.88, 0.94)	0.92 (0.89, 0.95)	0.99 (0.99, 0.99)	8.1 (5.3, 13.3)
WBC count	0.91 (0.88, 0.94)	0.92 (0.89, 0.95)	0.99 (0.99, 0.99)	10.7 (7.2, 17.6)
Total cholesterol	0.91 (0.88, 0.94)	0.92 (0.89, 0.95)	0.99 (0.99, 0.99)	12.4 (8.5, 18.6)
LDL cholesterol	0.91 (0.89, 0.94)	0.92 (0.90, 0.95)	0.99 (0.99, 0.99)	12.0 (8.2, 18.5)
Glucose	0.91 (0.89, 0.95)	0.92 (0.89, 0.95)	1.00 (1.00, 1.00)	1.3 (0.1, 2.8)
All five	0.91 (0.89, 0.95)	0.94 (0.91, 0.97)	0.97 (0.97, 0.98)	27.1 (19.8, 47.5)

*Note:* The mediators were modelled as continuous variables (per standard deviation increase). All the models were adjusted for age, sex, height, body mass index, baseline assessment centre, ethnicity, education, deprivation, smoking, alcohol consumption, physical activity, family history of cancer, and grip strength.

Abbreviations: CI, confidence interval; CRP, C‐reactive protein; HR, hazard ratio; LDL, low‐density lipoprotein; WBC, white blood cell.

^a^ 95% CIs for the total effect were estimated using bootstrapping (*n* = 200) and were slightly different from that in Table 2.

^b^ Pure natural direct effect represents the association between walking speed and any cancer that is independent of the mediator(s).

^c^ Total natural indirect effect represents the association between walking speed and any cancer that is mediated by the mediator(s).

^d^ Calculated as the ratio of total natural indirect effect to total effect.

### 3.4 Subgroup and Sensitivity Analyses

Subgroup analyses were performed to examine potential effect modification. In the UK Biobank, the association between walking speed and any cancer appeared to be stronger in men, white participants, normal‐weight individuals with a BMI < 25, ever‐smokers, and physically inactive individuals (*p* for interaction < 0.05) (Table S5). Similar results were found in the HKOS, where the association between walking speed and any cancer tended to be stronger in older adults aged ≥65 years and in men (*p* for interaction < 0.001) (Table S6). For site‐specific cancers, the association between walking speed and lung cancer in the UK Biobank was stronger in normal‐weight and physically inactive individuals and was statistically significant only in ever‐smokers but not never‐smokers (*p* for interaction < 0.05). For prostate cancer, we observed an interaction between age and walking pace such that the association was significant only in younger adults aged < 65 years (*p* for interaction = 0.003) (Table S5).

In the sensitivity analysis, the association between walking speed and prostate cancer was slightly attenuated when additionally adjusted for serum testosterone level and diabetes (HR for brisk vs. slow pace = 1.10, 95% CI = 1.01–1.21) (Table S7). Moreover, excluding the first 3 years of follow‐up had minimal impact on the association between walking speed and any cancer, lung cancer, and prostate cancer in the UK Biobank (Table S8), as well as between walking speed and any cancer in the HKOS (Table S9).

## 4 Discussion

To the best of our knowledge, this study is the largest to date that has investigated the relationship between walking speed and the risk of cancer. Using data from the population‐based UK Biobank cohort, we showed that a faster walking speed was associated with a decreased risk of developing any cancer and lung cancer, even after adjusting for multiple cancer risk factors including physical activity level and grip strength. This association tended to be stronger in men, white participants, individuals with a normal weight, ever‐smokers, and those who are physically inactive. Importantly, we observed largely consistent results in a Chinese population with walking speed measured using a timed walk test, further supporting the generalizability of this finding. In addition to the observed associations, our mediation analyses showed that the levels of CRP, WBC count, total cholesterol, and LDL cholesterol partially mediated the relationship between walking speed and cancer risk, suggesting inflammation and lipid metabolism dysfunction as the potential underlying mechanisms.

Despite a well‐established link between physical activity, including walking, and cancer risks [2, 3, 5], research on the association between walking speed and cancer has been scarce. Our findings align with a recent study in the UK Biobank, which similarly found that individuals with a self‐reported brisk walking pace had a longer lifetime lived without cancer or cardiovascular disease [29]. Notably, the association between walking speed and any cancer persisted regardless of whether walking speed was self‐reported in the UK Biobank or objectively measured in the HKOS. This suggests that the observed association may not be only explained by subjective health or psychological factors [30], but could also involve a potential physiological link between actual walking speed and cancer, which is likely to be complex. The ability to walk relies on the coordination and function of multiple physiological systems, including the nervous system, perceptual system, muscles, bone and joints, and energy production and delivery [31]. Hence, reduced walking speed may be an early indicator of a multisystem dysregulation, reflecting functional decline [7], sarcopenia [10], frailty [11], and biological aging [32], all of which have also been associated with cancer risks [24, 33, 34], and the decline in these systems could happen well before the incident cancer.

Several biological mechanisms may mediate the association between walking speed and cancer. Gait speed is an important marker of sarcopenia [10], and skeletal muscle is an endocrine organ that releases myokines (e.g. interleukin‐6, brain‐derived neurotrophic factor, tumour necrosis factor) to regulate inflammatory and metabolic pathways [12]. Increasing research has shown a link between sarcopenia and physical inactivity with cancer risk, largely involving a chronic inflammatory environment that promotes cancer cell growth and spread [12, 35]. Furthermore, sarcopenia is closely associated with metabolic disorders [12, 36], such as insulin resistance and lipid metabolism dysfunction, which are also linked to cancer development [14]. Additionally, sarcopenia is related to a decline in immune system function. As muscle mass decreases, the body's immune capacity weakens, particularly affecting the function of T cells and natural killer cells, thereby reducing the ability of sarcopenic individuals to effectively combat cancer [37]. Therefore, the reduced cancer risk among fast walkers could be at least partially explained by an overall lower inflammation and improved immune and metabolic functions. This was also supported by our mediation analyses, which showed that CRP, WBC count, total cholesterol, LDL cholesterol, and glucose levels together mediated around one‐fourth of the association between walking speed and cancer risk.

When stratified by cancer types in the UK Biobank, we found that a faster walking speed was associated with a significantly reduced risk of lung cancer. This association also remained essentially unchanged when we removed the first 3 years of follow‐up, and thus, it is unlikely explained by reverse causation. This is consistent with the findings from a recent Mendelian randomization study, which suggested that genetically predicted walking frequency and pace are associated with lower risk of lung cancer [17]. The strong association between walking speed and lung cancer could be explained by the improved cardiorespiratory fitness in brisk walkers that reduces their risk of developing lung cancer [38]. Moreover, treadmill training can particularly improve lung function among various exercises [39]. However, similar to previous findings on physical activity and lung cancer [2], this association was statistically significant only for ever‐smokers but not never‐smokers. This could be partly due to residual confounding by smoking habits, or that the pathogenesis of lung cancer is different in smokers versus never‐smokers [2].

On the other hand, we found a somewhat unexpected increased risk of prostate cancer associated with a brisk walking pace in the UK Biobank, although this association was not statistically significant after correction for multiple testing. Similar findings have also been reported in the literature, where leisure‐time physical activity has been linked to a higher incidence of prostate cancer [2]. Similarly, physical frailty [33] and higher biological age [34] have been associated with a lower incidence of prostate cancer. The observed association for prostate cancer may be influenced by a screening bias, as younger and healthier men are more likely to attend prostate‐specific antigen screening tests, leading to a higher rate of detecting nonadvanced prostate cancer [2]. This was also seen from our subgroup analysis, where we found an effect modification by age and a significant association between walking pace and prostate cancer only in younger adults, but not in older adults aged 65 years or above.

### 4.1 Strengths and Limitations

The main strengths of this study include the use of two diverse prospective cohorts with multiple ethnic groups, a relatively long follow‐up period, and the inclusion of both self‐reported and timed measures of walking speed to validate our findings. The large sample size in the UK Biobank also allowed adequate statistical power to examine various cancer subtypes as well as conducting subgroup analyses. However, it is important to acknowledge several limitations. The UK Biobank is a relatively healthy cohort with lower cancer incidence compared to the general UK population [40]. Similarly, the HKOS primarily consists of women and healthy individuals without major diseases affecting bone and mineral metabolism [19]. As such, our findings may not be fully generalizable to the general population. Besides, due to the relatively small sample size in the HKOS, there were only a few incident cases for specific cancers such as colorectal, prostate, and stomach cancer, and we lack statistical power to perform additional subgroup and threshold effect analyses in the HKOS. Further studies are therefore needed to replicate the observed association between walking speed and specific cancers, and test the optimal cut‐off point for walking speed that can best predict cancer risks. Finally, as in other observational studies, while we have controlled for a range of sociodemographic and lifestyle factors in our analysis, our results could still be biased by unmeasured and residual confounding and therefore do not infer causality.

## 5 Conclusion

The present study provides new evidence that a faster walking speed, whether subjectively reported or objectively measured, is associated with a reduced risk of developing any cancer and lung cancer, independently of physical activity level and grip strength. Biomarkers of inflammation and lipid metabolism mediated approximately one‐fourth of the association, indicating that lower inflammation and improved lipid profiles may be potential mechanisms mediating this relationship. While further research is needed to replicate these findings in diverse populations, our results suggest that, in addition to emphasizing on the amount or duration of walking, increasing walking speed could provide additional benefits in reducing cancer risk, potentially by improving immune and metabolic functions.

## Author Contributions

JKLM and CLC contributed to the conception and design of the study. JKLM performed the statistical analysis and drafted the manuscript. KCBT, JJ, SH, and CLC contributed to acquisition of the data. All authors contributed to interpretation of the data and critically revised the manuscript for important intellectual content. All authors read and approved the final version of the manuscript.

## Conflicts of Interest

The authors declare no conflicts of interest.

## Supporting information


**Figure S1.** Flowchart of sample selection.
**Figure S2.** Dose–response relationship between walking speed and the risk of any cancer in the Hong Kong Osteoporosis Study. The black solid line in the upper panel represents hazard ratios and the corresponding 95% confidence intervals (shaded areas) estimated using restricted cubic spline Cox regression models with knots placed at the 1st quartile (1.07 m/s), median (1.22 m/s), and 3rd quartile (1.35 m/s). Walking speed of 1.0 m/s was used as the reference value. The model was adjusted for age, sex, height, body mass index, smoking status, alcohol consumption, education level, physical activity, family history of cancer, and grip strength. The lower panel shows the distribution of walking speed in HKOS.
**Table S1.** List of the ICD codes used to define cancer cases.
**Table S2.** Baseline characteristics stratified by walking speed.
**Table S3.** Baseline characteristics stratified by cancer status during follow‐up.
**Table S4.** Mediation analysis for the association between self‐reported walking speed and lung cancer, for biomarkers of inflammation and lipid metabolism in the UK Biobank.
**Table S5.** Subgroup analysis for the association between walking speed and risk of any cancer, lung cancer, and prostate cancer in the UK Biobank.
**Table S6.** Subgroup analysis for the association between walking speed and risk of any cancer in the Hong Kong Osteoporosis Study.
**Table S7.** Association between walking speed and prostate cancer in the UK Biobank when further adjusted for testosterone and diabetes.
**Table S8.** Association between walking speed and risk of any caner, lung cancer, and prostate cancer after excluding the first 3 years of follow‐up in the UK Biobank.
**Table S9.** Association between walking speed and risk of any caner after excluding the first 3 years of follow‐up in the Hong Kong Osteoporosis Study.

## Data Availability

The UK Biobank is an open access resource. All bona fide researchers can apply to use its data for health‐related research that is in the public interest (http://www.ukbiobank.ac.uk/register‐apply). The Hong Kong Osteoporosis Study cannot be made publicly available due to The Personal Data (Privacy) Ordinance (Cap. 486) in Hong Kong as stated in the informed consent forms. For other requests regarding data, please contact the corresponding author (lung1212@hku.hk).
