# Supplementary material for: Walking Speed and Risk of Cancer in Two Prospective Cohort Studies
Source: J Cachexia Sarcopenia Muscle. 2025 Apr 24;16(3):e13792. doi: 10.1002/jcsm.13792 (PMC12022227; doi:10.1002/jcsm.13792)
Supplement: Supplementary file 1 — Figure S1. Flowchart of sample selection. Figure S2. Dose–response relationship between walking speed and the risk of any cancer in the Hong Kong Osteoporosis Study. The black solid line in the upper panel represents hazard ratios and the corresponding 95% confidence intervals (shaded areas) estimated using restricted cubic spline Cox regression models with knots placed at the 1st quartile (1.07 m/s), median (1.22 m/s), and 3rd quartile (1.35 m/s). Walking speed of 1.0 m/s was used as the reference value. The model was adjusted for age, sex, height, body mass index, smoking status, alcohol consumption, education level, physical activity, family history of cancer, and grip strength. The lower panel shows the distribution of walking speed in HKOS. Table S1. List of the ICD codes used to define cancer cases. Table S2. Baseline characteristics stratified by walking speed. Table S3. Baseline characteristics stratified by cancer status during follow‐up. Table S4. Mediation analysis for the association between self‐reported walking speed and lung cancer, for biomarkers of inflammation and lipid metabolism in the UK Biobank. Table S5. Subgroup analysis for the association between walking speed and risk of any cancer, lung cancer, and prostate cancer in the UK Biobank. Table S6. Subgroup analysis for the association between walking speed and risk of any cancer in the Hong Kong Osteoporosis Study. Table S7. Association between walking speed and prostate cancer in the UK Biobank when further adjusted for testosterone and diabetes. Table S8. Association between walking speed and risk of any caner, lung cancer, and prostate cancer after excluding the first 3 years of follow‐up in the UK Biobank. Table S9. Association between walking speed and risk of any caner after excluding the first 3 years of follow‐up in the Hong Kong Osteoporosis Study. [file JCSM-16-e13792-s001.docx]

**Supplementary Material**

[Supplementary Figure 1. Flowchart of sample selection. 2](#_Toc183446411)

[Supplementary Figure 2. Dose-response relationship between walking speed and the risk of any cancer in the Hong Kong Osteoporosis Study. 3](#_Toc183446412)

[Supplementary Table 1. List of the ICD codes used to define cancer cases. 4](#_Toc183446413)

[Supplementary Table 2. Baseline characteristics stratified by walking speed. 5](#_Toc183446414)

[Supplementary Table 3. Baseline characteristics stratified by cancer status during follow-up. 7](#_Toc183446415)

[Supplementary Table 4. Mediation analysis for the association between self-reported walking speed and lung cancer, for biomarkers of inflammation and lipid metabolism in the UK Biobank. 9](#_Toc183446416)

[Supplementary Table 5. Subgroup analysis for the association between walking speed and risk of any cancer, lung cancer, and prostate cancer in the UK Biobank. 10](#_Toc183446417)

[Supplementary Table 6. Subgroup analysis for the association between walking speed and risk of any cancer in the Hong Kong Osteoporosis Study. 12](#_Toc183446418)

[Supplementary Table 7. Association between walking speed and prostate cancer in the UK Biobank when further adjusted for testosterone and diabetes. 13](#_Toc183446419)

[Supplementary Table 8. Association between walking speed and risk of any caner, lung cancer, and prostate cancer after excluding the first 3 years of follow-up in the UK Biobank. 14](#_Toc183446420)

[Supplementary Table 9. Association between walking speed and risk of any caner after excluding the first 3 years of follow-up in the Hong Kong Osteoporosis Study 15](#_Toc183446421)

**
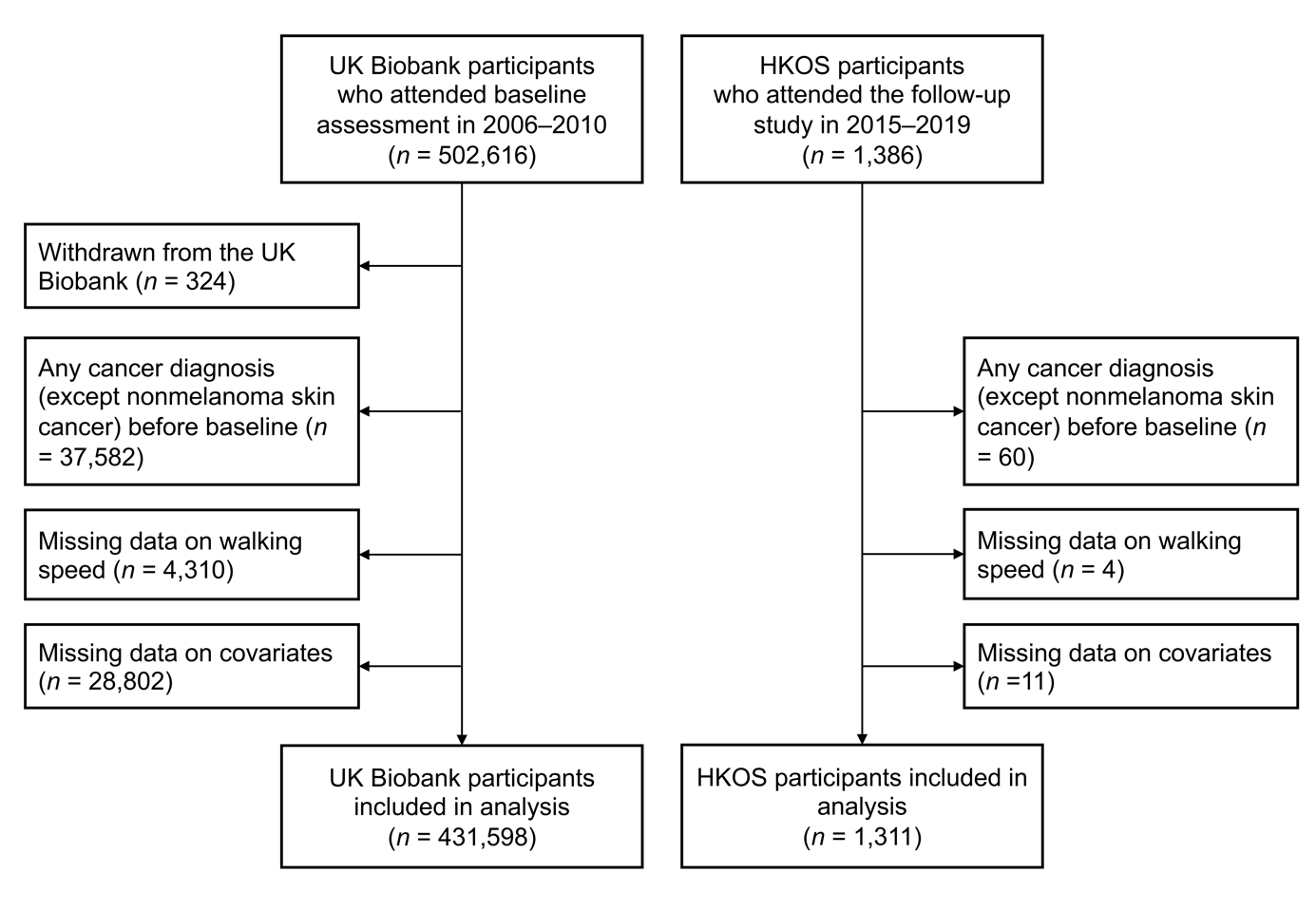
**

# **Supplementary Figure 1.** Flowchart of sample selection.


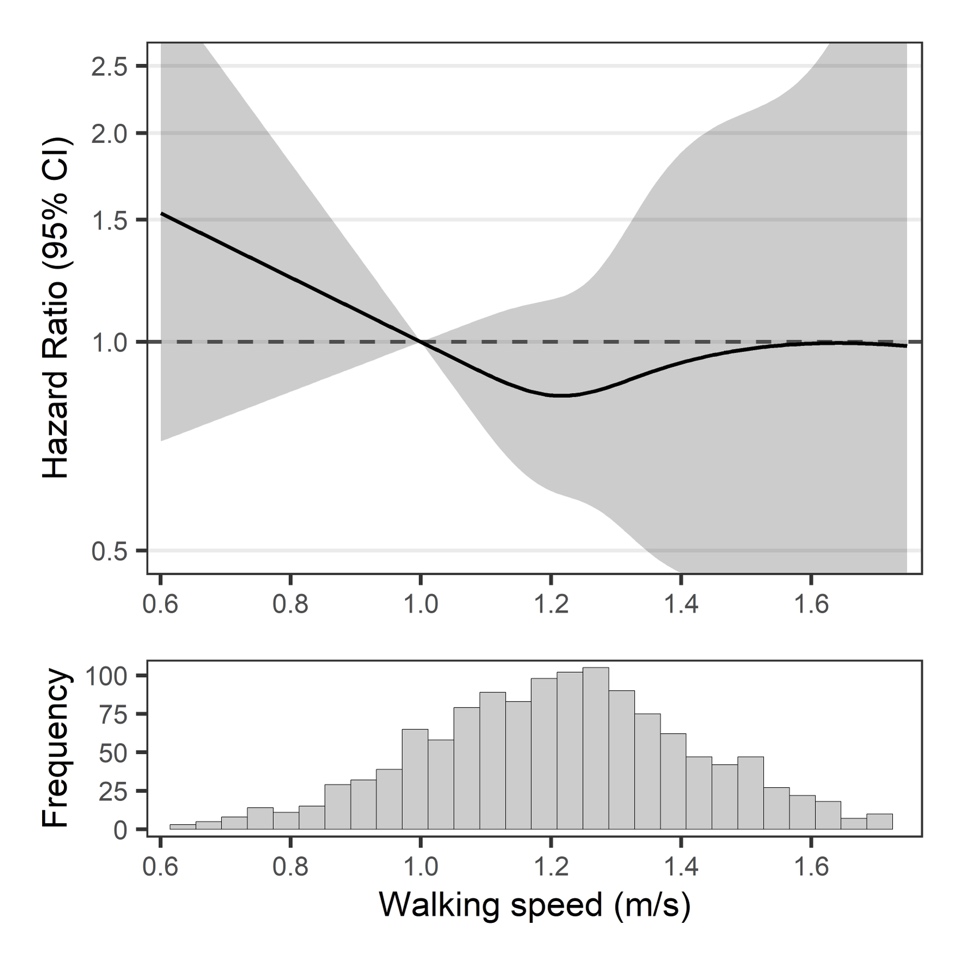


# **Supplementary Figure 2.** Dose-response relationship between walking speed and the risk of any cancer in the Hong Kong Osteoporosis Study.

The black solid line in the upper panel represents hazard ratios and the corresponding 95% confidence intervals (shaded areas) estimated using restricted cubic spline Cox regression models with knots placed at the 1^st^ quartile (1.07 m/s), median (1.22 m/s), and 3^rd^ quartile (1.35 m/s). Walking speed of 1.0 m/s was used as the reference value. The model was adjusted for age, sex, height, body mass index, smoking status, alcohol consumption, education level, physical activity, family history of cancer, and grip strength. The lower panel shows the distribution of walking speed in HKOS.

# **Supplementary Table 1.** List of the ICD codes used to define cancer cases.

| **Cancer site** | **ICD-9** | **ICD-10** |
| --- | --- | --- |
| Any cancer (except non-melanoma skin) | 140–208, except 173 | C00–97, except C44 |
| Lung cancer (including trachea) | 162 | C33–34 |
| Breast cancer in women | 174 | C50 |
| Colorectal cancer | 153–154 | C18–20 |
| Prostate cancer in men | 185 | C61 |
| Stomach cancer | 151 | C16 |

*ICD*, International Classification of Diseases.

# **Supplementary Table 2.** Baseline characteristics stratified by walking speed.

| Characteristic | **UK Biobank** | | | | **HKOS** | | |
| --- | --- | --- | --- | --- | --- | --- | --- |
|  | Slow | Average | Brisk | *p*^a^ | <1.0 m/s | ≥1.0 m/s | *p*^a^ |
| Number of individuals | 32,298 (7.5) | 226,207 (52.4) | 173,093 (40.1) | - | 210 (16.0) | 1,101 (84.0) | - |
| Age, year, mean (SD) | 58.6 (7.6) | 56.9 (8.0) | 55.0 (8.1) | <0.001 | 67.0 (9.7) | 56.1 (11.4) | <0.001 |
| Sex, n (%) |  |  |  |  |  |  | 0.292 |
| Women | 17,483 (54.1) | 120,705 (53.4) | 91,167 (52.7) | <0.001 | 172 (81.9) | 863 (78.4) |  |
| Men | 14,815 (45.8) | 105,502 (46.6) | 81,926 (47.3) | <0.001 | 38 (18.1) | 238 (21.6) |  |
| Height, m, mean (SD) | 1.66 (0.09) | 1.68 (0.09) | 1.70 (0.09) | <0.001 | 1.55 (0.08) | 1.59 (0.08) | <0.001 |
| BMI, kg/m^2^, mean (SD) | 31.3 (6.6) | 28.0 (4.7) | 25.8 (3.8) | <0.001 | 24.1 (4.3) | 23.1 (3.5) | 0.001 |
| Baseline assessment center, n (%) |  |  |  | <0.001 |  |  | - |
| England | 28,395 (87.9) | 200,849 (88.8) | 153,147 (88.5) |  | - | - |  |
| Wales | 1499 (4.6) | 9460 (4.2) | 7005 (4.0) |  | - | - |  |
| Scotland | 2404 (7.4) | 15,898 (7.0) | 12,941 (7.5) |  | - | - |  |
| Ethnicity, n (%) |  |  |  | <0.001 |  |  | - |
| White | 29,077 (90.0) | 212,778 (94.1) | 167,391 (96.7) |  | - | - |  |
| Asian | 1,503 (4.7) | 5,914 (2.6) | 1,953 (1.1) |  | 210 (100) | 1,101 (100) |  |
| Black | 872 (2.7) | 3991 (1.8) | 1815 (1.0) |  | - | - |  |
| Others | 846 (2.6) | 3524 (1.6) | 1934 (1.1) |  | - | - |  |
| Education, n (%) |  |  |  | <0.001 |  |  | <0.001 |
| Primary or below | 10,767 (33.3) | 40,992 (18.1) | 17,496 (10.1) |  | 81 (38.6) | 146 (13.3) |  |
| Secondary | 15,018 (46.5) | 117,368 (51.9) | 84,262 (48.7) |  | 95 (45.2) | 579 (52.6) |  |
| College or university | 6513 (20.2) | 67,847 (30.0) | 71,335 (41.2) |  | 34 (16.2) | 376 (34.2) |  |
| Deprivation index, mean (SD) | -0.04 (3.52) | -1.35 (3.05) | -1.63 (2.90) | <0.001 | - | - | - |
| Smoking status, n (%) |  |  |  | <0.001 |  |  | 0.021 |
| Never | 14,396 (44.6) | 122,814 (54.3) | 101,235 (58.5) |  | 201 (95.7) | 1034 (93.9) |  |
| Previous | 12,272 (38.0) | 79,354 (35.1) | 57,059 (33.0) |  | 1 (0.5) | 42 (3.8) |  |
| Current | 5630 (17.4) | 24,039 (10.6) | 14,799 (8.5) |  | 8 (3.8) | 25 (2.3) |  |

**Supplementary Table 2.** (*continued*).

| Characteristic | **UK Biobank** | | | | **HKOS** | | |
| --- | --- | --- | --- | --- | --- | --- | --- |
|  | Slow | Average | Brisk | *p* | <1.0 m/s | ≥1.0 m/s | *p* |
| Alcohol consumption, n (%) |  |  |  | <0.001 |  |  | 0.008 |
| Never | 2889 (9.0) | 9894 (4.4) | 5122 (3.0) |  | 157 (74.8) | 704 (63.9) |  |
| Occasionally | 9045 (28.0) | 34,408 (15.2) | 19,004 (11.0) |  | 21 (10.0) | 147 (13.4) |  |
| Once a month | 3853 (11.9) | 26,362 (11.7) | 17,781 (10.3) |  | 21 (10.0) | 121 (11.0) |  |
| Once a week | 16,511 (51.1) | 155,543 (68.8) | 131,186 (75.8) |  | 11 (5.2) | 129 (11.7) |  |
| Physical activity, MET-min/week, mean (SD) | 1,499.7 (2,713.1) | 2,705.9 (3,764.6) | 3,138.7 (4,023.6) | <0.001 | 2,317.8 (2,601.9) | 2,817.3 (3,024.6) | 0.025 |
| Family history of cancer, n (%) | 11,610 (35.9) | 79,064 (35.0) | 59,153 (34.2) | <0.001 | 55 (26.2) | 287 (26.1) | 1 |
| Grip strength, kg, mean (SD) | 26.1 (11.2) | 30.6 (10.9) | 32.5 (10.9) | <0.001 | 19.7 (6.4) | 24.7 (7.9) | <0.001 |
| CRP, mg/L, mean (SD) | 4.62 (6.30) | 2.68 (4.25) | 1.90 (3.45) | <0.001 | - | - | - |
| WBC count, 10^9^ cells/L, mean (SD) | 7.60 (2.13) | 6.96 (1.86) | 6.60 (1.75) | <0.001 | - | - | - |
| Total cholesterol, mmol/L, mean (SD) | 5.41 (1.24) | 5.70 (1.15) | 5.74 (1.09) | <0.001 | 4.99 (0.94) | 5.06 (0.91) | 0.308 |
| LDL cholesterol, mmol/L, mean (SD) | 3.38 (0.94) | 3.57 (0.87) | 3.57 (0.84) | <0.001 | 2.81 (0.80) | 2.86 (0.81) | 0.378 |
| Glucose, mmol/L, mean (SD) | 5.50 (1.92) | 5.14 (1.26) | 5.01 (0.96) | <0.001 | 5.33 (0.99) | 4.98 (0.92) | <0.001 |
| Any incident cancer, n (%) | 4768 (14.8) | 27,670 (12.2) | 18,042 (10.4) | <0.001 | 21 (10.0) | 45 (4.1) | 0.001 |
| Died during follow-up, n (%) | 4345 (13.5) | 11,401 (5.0) | 5662 (3.3) | <0.001 | 13 (6.2) | 12 (1.1) | <0.001 |

*BMI*, body mass index; *CRP*, C-reactive protein; *LDL*, low-density lipoprotein; *MET*, metabolic equivalent of task; *HKOS*, Hong Kong Osteoporosis Study; *WBC*, white blood cell.

1. *P*-values were based on ANOVA or t-tests for continuous variables and chi-squared tests for categorical variables.

# **Supplementary Table 3.** Baseline characteristics stratified by cancer status during follow-up.

| Characteristic | **UK Biobank** | | | **HKOS** | | |
| --- | --- | --- | --- | --- | --- | --- |
|  | No cancer | Incident cancer | *p*^a^ | No cancer | Incident cancer | *p*^a^ |
| Number of individuals | 381,118 (88.3) | 50,480 (11.7) | - | 1,245 (95.0) | 66 (5.0) | - |
| Age, year, mean (SD) | 55.8 (8.1) | 59.7 (7.1) | <0.001 | 57.5 (11.9) | 63.1 (9.7) | <0.001 |
| Sex, n (%) |  |  | <0.001 |  |  | 0.666 |
| Women | 205,340 (53.9) | 24,015 (47.6) |  | 981 (78.8) | 54 (81.8) |  |
| Men | 175,778 (46.1) | 26,465 (52.4) |  | 264 (21.2) | 12 (18.2) |  |
| Height, m, mean (SD) | 1.69 (0.09) | 1.69 (0.09) | <0.001 | 1.58 (0.08) | 1.57 (0.07) | 0.313 |
| BMI, kg/m^2^, mean (SD) | 27.33 (4.74) | 27.73 (4.76) | <0.001 | 23.26 (3.66) | 23.78 (3.90) | 0.268 |
| Baseline assessment center, n (%) |  |  | <0.001 |  |  | - |
| England | 337,804 (88.6) | 44,587 (88.3) |  | - | - |  |
| Wales | 16,399 (4.3) | 1565 (3.1) |  | - | - |  |
| Scotland | 26,915 (7.1) | 4328 (8.6) |  | - | - |  |
| Ethnicity, n (%) |  |  | <0.001 |  |  | - |
| White | 360,492 (94.6) | 48,754 (96.6) |  | - | - |  |
| Asian | 6115 (1.6) | 563 (1.1) |  | 1,245 (100) | 66 (100) |  |
| Black | 8736 (2.3) | 634 (1.3) |  | - | - |  |
| Others | 5775 (1.5) | 529 (1.0) |  | - | - |  |
| Education, n (%) |  |  | <0.001 |  |  | 0.617 |
| Primary or below | 58,709 (15.4) | 10,546 (20.9) |  | 213 (17.1) | 14 (21.2) |  |
| Secondary | 192,312 (50.5) | 24,336 (48.2) |  | 640 (51.4) | 34 (51.5) |  |
| College or university | 130,097 (34.1) | 15,598 (30.9) |  | 392 (31.5) | 18 (27.3) |  |
| Deprivation index, mean (SD) | -1.36 (3.05) | -1.38 (3.07) | 0.126 |  |  |  |
| Smoking status, n (%) |  |  | <0.001 |  |  | 0.459 |
| Never | 214,131 (56.2) | 24,314 (48.2) |  | 1175 (94.4) | 60 (90.9) |  |
| Previous | 128,751 (33.8) | 19,934 (39.5) |  | 40 (3.2) | 3 (4.5) |  |
| Current | 38,236 (10.0) | 6232 (12.3) |  | 30 (2.4) | 3 (4.5) |  |

**Supplementary Table 3.** (*continued*).

| Characteristic | **UK Biobank** | | | **HKOS** | | |
| --- | --- | --- | --- | --- | --- | --- |
|  | No cancer | Incident cancer | *p*^a^ | No cancer | Incident cancer | *p*^a^ |
| Alcohol consumption, n (%) |  |  | <0.001 |  |  | 0.751 |
| Never | 16,009 (4.2) | 1896 (3.8) |  | 820 (65.9) | 41 (62.1) |  |
| Occasionally | 55,017 (14.4) | 7440 (14.7) |  | 157 (12.6) | 11 (16.7) |  |
| Once a month | 42,833 (11.2) | 5163 (10.2) |  | 134 (10.8) | 8 (12.1) |  |
| Once a week | 267,259 (70.1) | 35,981 (71.3) |  | 134 (10.8) | 6 (9.1) |  |
| Physical activity, MET-min/week, mean (SD) | 2,793.4 (3,832.7) | 2,757.1 (3,786.7) | 0.045 | 2,719.8 (2,899.4) | 3,066.8 (4,031.4) | 0.355 |
| Family history of cancer, n (%) | 130,133 (34.1) | 19,694 (39.0) | <0.001 | 326 (26.2) | 16 (24.2) | 0.837 |
| Grip strength, kg, mean (SD) | 31.04 (11.07) | 31.11 (10.74) | 0.161 | 23.97 (7.93) | 21.77 (6.63) | 0.027 |
| CRP, mg/L, mean (SD) | 2.47 (4.15) | 2.82 (4.54) | <0.001 | - | - | - |
| WBC count, 10^9^ cells/L, mean (SD) | 6.83 (1.78) | 7.09 (2.39) | <0.001 | - | - | - |
| Total cholesterol, mmol/L, mean (SD) | 5.70 (1.13) | 5.62 (1.17) | <0.001 | 5.06 (0.92) | 4.93 (0.91) | 0.280 |
| LDL cholesterol, mmol/L, mean (SD) | 3.56 (0.86) | 3.51 (0.88) | <0.001 | 2.86 (0.81) | 2.68 (0.76) | 0.090 |
| Glucose, mmol/L, mean (SD) | 5.10 (1.21) | 5.18 (1.30) | <0.001 | 5.03 (0.95) | 5.13 (0.74) | 0.441 |
| Died during follow-up, n (%) | 9411 (2.5) | 11,997 (23.8) | <0.001 | 16 (1.3) | 9 (13.6) | <0.001 |

*BMI*, body mass index; *CRP*, C-reactive protein; *LDL*, low-density lipoprotein; *MET*, metabolic equivalent of task; *HKOS*, Hong Kong Osteoporosis Study; *WBC*, white blood cell.

1. *P*-values were based on t-tests for continuous variables and chi-squared tests for categorical variables.

# **Supplementary Table 4.** Mediation analysis for the association between self-reported walking speed and lung cancer, for biomarkers of inflammation and lipid metabolism in the UK Biobank.

| Mediators | Total effect^a^,  HR (95% CI) | Pure natural direct effect^b^,  HR (95% CI) | Total natural indirect effect^c^,  HR (95% CI) | Proportion mediated^d^,  % (95% CI) |
| --- | --- | --- | --- | --- |
| Brisk vs. slow | | | | |
| CRP | 0.46 (0.41, 0.52) | 0.47 (0.42, 0.53) | 0.97 (0.97, 0.98) | 2.3 (1.6, 3.0) |
| WBC count | 0.47 (0.42, 0.52) | 0.48 (0.43, 0.53) | 0.98 (0.97, 0.98) | 2.0 (1.6, 2.7) |
| Total cholesterol | 0.46 (0.41, 0.52) | 0.47 (0.42, 0.53) | 0.97 (0.97, 0.98) | 2.3 (1.5, 3.2) |
| LDL cholesterol | 0.46 (0.41, 0.52) | 0.47 (0.42, 0.53) | 0.98 (0.97, 0.98) | 2.2 (1.4, 3.2) |
| Glucose | 0.45 (0.40, 0.51) | 0.46 (0.40, 0.51) | 1.00 (0.99, 1.00) | 0.2 (-0.3, 0.6) |
| All five | 0.45 (0.41, 0.52) | 0.49 (0.44, 0.56) | 0.93 (0.92, 0.95) | 5.9 (4.5, 7.9) |
| Average vs. slow | | | | |
| CRP | 0.61 (0.55, 0.68) | 0.62 (0.56, 0.69) | 0.98 (0.97, 0.98) | 3.4 (2.6, 4.6) |
| WBC count | 0.61 (0.55, 0.67) | 0.62 (0.56, 0.69) | 0.99 (0.98, 0.99) | 2.3 (1.8, 3.3) |
| Total cholesterol | 0.61 (0.56, 0.67) | 0.63 (0.57, 0.69) | 0.98 (0.97, 0.98) | 3.8 (2.5, 5.4) |
| LDL cholesterol | 0.61 (0.55, 0.67) | 0.63 (0.56, 0.69) | 0.98 (0.97, 0.98) | 3.9 (2.5, 5.7) |
| Glucose | 0.61 (0.55, 0.68) | 0.61 (0.56, 0.68) | 1.00 (0.99, 1.00) | 0.4 (-0.3, 1.1) |
| All five | 0.60 (0.54, 0.66) | 0.63 (0.57, 0.70) | 0.95 (0.94, 0.96) | 8.0 (5.7, 11.1) |

*CI*, confidence interval; *CRP*, C-reactive protein; *HR*, hazard ratio; *LDL*, low-density lipoprotein; *WBC*, white blood cell. The mediators were modeled as continuous variables (per standard deviation increase). All the models were adjusted for age, sex, height, body mass index, baseline assessment center, ethnicity, education, deprivation, smoking, alcohol consumption, physical activity, family history of cancer, and grip strength.

1. 95% CIs for the total effect were estimated using bootstrapping (n=200) and were slightly different from that in Table 2.
2. Pure natural direct effect represents the association between walking speed and lung cancer that is independent of the mediator(s).
3. Total natural indirect effect represents the association between walking speed and lung cancer that is mediated by the mediator(s).
4. Calculated as the ratio of total natural indirect effect to total effect.

# **Supplementary Table 5**. Subgroup analysis for the association between walking speed and risk of any cancer, lung cancer, and prostate cancer in the UK Biobank.

| Subgroup | Self-reported walking speed, HR (95% CI) | | | *P*_interaction_^a^ |
| --- | --- | --- | --- | --- |
|  | Slow | Average | Brisk |  |
| **Any cancer** | | | | |
| Age |  |  |  |  |
| <65 years (n=353,400) | 1 (ref) | 0.93 (0.89, 0.97)** | 0.89 (0.85, 0.93)** | 0.060 |
| ≥65 years (n=78,198) | 1 (ref) | 0.89 (0.85, 0.94)** | 0.84 (0.79, 0.89)** |  |
| Sex |  |  |  |  |
| Women (n=229,355) | 1 (ref) | 0.92 (0.88, 0.96)** | 0.89 (0.85, 0.94)** | <0.001 |
| Men (n=202,243) | 1 (ref) | 0.92 (0.88, 0.96)** | 0.87 (0.83, 0.92)** |  |
| Ethnicity |  |  |  |  |
| White (n=409,246) | 1 (ref) | 0.92 (0.89, 0.95)** | 0.87 (0.84, 0.90)** | <0.001 |
| Non-white (n=22,352) | 1 (ref) | 0.85 (0.74, 0.98)* | 0.95 (0.81, 1.12) |  |
| Body mass index |  |  |  |  |
| <25 kg/m^2^ (n=143,665) | 1 (ref) | 0.77 (0.71, 0.83)** | 0.74 (0.68, 0.80)** | <0.001 |
| ≥25 kg/m^2^ (n=288,933) | 1 (ref) | 0.92 (0.89, 0.95)** | 0.87 (0.84, 0.91)** |  |
| Smoking status |  |  |  |  |
| Never-smoker (n=238,445) | 1 (ref) | 0.96 (0.91, 1.02) | 0.94 (0.89, 0.99)* | <0.001 |
| Ever-smoker (n=193,153) | 1 (ref) | 0.87 (0.84, 0.91)** | 0.81 (0.77, 0.85)** |  |
| Physical activity |  |  |  |  |
| Low (n=98,224) | 1 (ref) | 0.89 (0.85, 0.94)** | 0.87 (0.82, 0.93)** | <0.001 |
| Moderate/high (n=333,374) | 1 (ref) | 0.94 (0.90, 0.98)** | 0.90 (0.86, 0.94)** |  |
| **Lung cancer** | | | | |
| Age |  |  |  |  |
| <65 years (n=353,400) | 1 (ref) | 0.61 (0.54, 0.69)** | 0.48 (0.41, 0.55)** | 0.14 |
| ≥65 years (n=78,198) | 1 (ref) | 0.65 (0.56, 0.76)** | 0.47 (0.39, 0.57)** |  |
| Sex |  |  |  |  |
| Women (n=229,355) | 1 (ref) | 0.59 (0.52, 0.68)** | 0.49 (0.42, 0.58)** | 0.08 |
| Men (n=202,243) | 1 (ref) | 0.66 (0.58, 0.75)** | 0.47 (0.40, 0.54)** |  |
| Ethnicity |  |  |  |  |
| White (n=409,246) | 1 (ref) | 0.63 (0.57, 0.69)** | 0.48 (0.42, 0.53)** | 0.10 |
| Non-white (n=22,352) | 1 (ref) | 0.49 (0.29, 0.82)** | 0.49 (0.26, 0.93)* |  |
| Body mass index |  |  |  |  |
| <25 kg/m^2^ (n=143,665) | 1 (ref) | 0.52 (0.44, 0.63)** | 0.41 (0.33, 0.50)** | 0.003 |
| ≥25 kg/m^2^ (n=288,933) | 1 (ref) | 0.71 (0.64, 0.79)** | 0.56 (0.49, 0.64)** |  |
| Smoking status |  |  |  |  |
| Never-smoker (n=238,445) | 1 (ref) | 0.85 (0.60, 1.19) | 0.77 (0.53, 1.11) | <0.001 |
| Ever-smoker (n=193,153) | 1 (ref) | 0.55 (0.50, 0.61)** | 0.36 (0.33, 0.42)** |  |
| Physical activity |  |  |  |  |
| Low (n=98,224) | 1 (ref) | 0.61 (0.53, 0.71)** | 0.47 (0.38, 0.58)** | 0.013 |
| Moderate/High (n=333,374) | 1 (ref) | 0.66 (0.58, 0.75)** | 0.50 (0.44, 0.58)** |  |

**Supplementary Table 5**. (*continued*).

| Subgroup | Self-reported walking speed, HR (95% CI) | | | *P*_interaction_^a^ |
| --- | --- | --- | --- | --- |
|  | Slow | Average | Brisk |  |
| **Prostate cancer in men** | | | | |
| Age |  |  |  |  |
| <65 years (n=162,664) | 1 (ref) | 1.17 (1.05, 1.32)** | 1.16 (1.04, 1.31)** | 0.003 |
| ≥65 years (n=39,579) | 1 (ref) | 1.02 (0.90, 1.15) | 1.06 (0.93, 1.22) |  |
| Body mass index |  |  |  |  |
| <25 kg/m^2^ (n=51,271) | 1 (ref) | 1.04 (0.84, 1.28) | 1.05 (0.85, 1.31) | 0.67 |
| ≥25 kg/m^2^ (n=150,972) | 1 (ref) | 1.14 (1.04, 1.24)** | 1.17 (1.07, 1.29)** |  |
| Ethnicity |  |  |  |  |
| White (n=191,937) | 1 (ref) | 1.12 (1.03, 1.22)** | 1.12 (1.03, 1.23)* | 0.05 |
| Non-white (n=10,306) | 1 (ref) | 0.90 (0.67, 1.22) | 1.11 (0.78, 1.57) |  |
| Smoking status |  |  |  |  |
| Never-smoker (n=100,120) | 1 (ref) | 1.09 (0.95, 1.25) | 1.09 (0.94, 1.25) | 0.52 |
| Ever-smoker (n=102,123) | 1 (ref) | 1.10 (1.00, 1.22) | 1.13 (1.02, 1.27)* |  |
| Physical activity |  |  |  |  |
| Low (n=43,808) | 1 (ref) | 1.15 (1.01, 1.31)* | 1.22 (1.05, 1.43)** | 0.13 |
| Moderate/High (n=158,435) | 1 (ref) | 1.08 (0.97, 1.20) | 1.08 (0.97, 1.21) |  |

*CI*, confidence interval; *HR*, hazard ratio. All the models were adjusted for age, sex, height, body mass index, baseline assessment center, ethnicity, education, deprivation, smoking, alcohol consumption, physical activity, family history of cancer, and grip strength. **p*<.05; ***p*<.01.

1. *P*_interaction_ was based on likelihood ratio tests comparing models with and without the interaction terms between walking speed and the subgroup indicator.
2. Physical activity was categorized based on the IPAQ criteria. A low physical activity is defined as not meeting the recommendation of performing moderate/vigorous/walking activities.

# **Supplementary Table 6**. Subgroup analysis for the association between walking speed and risk of any cancer in the Hong Kong Osteoporosis Study.

| Subgroup | Timed walking speed, HR (95% CI) | | *P*_interaction_^a^ |
| --- | --- | --- | --- |
|  | <1.0 m/s | ≥1.0 m/s |  |
| Age |  |  |  |
| <65 years (n=670) | 1 (ref) | 0.93 (0.17, 4.59) | <0.001 |
| ≥65 years (n=641) | 1 (ref) | 0.50 (0.26, 0.94)* |  |
| Sex |  |  |  |
| Women (n=1035) | 1 (ref) | 0.60 (0.31, 1.15) | <0.001 |
| Men (n=276) | 1 (ref) | 0.37 (0.10, 1.40) |  |

*CI*, confidence interval; *HR*, hazard ratio. All the models were adjusted for age, sex, height, body mass index, ethnicity, education, smoking, alcohol consumption, physical activity, family history of cancer, and grip strength. **p*<.05.

1. *P*_interaction_ was based on likelihood ratio tests comparing models with and without the interaction terms between walking speed and the subgroup indicator.

# **Supplementary Table 7.** Association between walking speed and prostate cancer in the UK Biobank when further adjusted for testosterone and diabetes.

| Walking speed | HR (95% CI) | *p* |
| --- | --- | --- |
| Slow | 1 (ref) | - |
| Average | 1.08 (1.00, 1.18) | 0.06 |
| Brisk | 1.10 (1.01, 1.21) | 0.035 |

*CI*, confidence interval; *HR*, hazard ratio. **p*<.05; ***p*<.01. The model was adjusted for age, sex, height, body mass index, baseline assessment center, ethnicity, education, deprivation, smoking, alcohol consumption, physical activity, and family history of cancer, grip strength, serum testosterone level, and diabetes. Due to missing data on testosterone and diabetes, the number of individuals in this analysis was n=189,762.

# **Supplementary Table 8.** Association between walking speed and risk of any caner, lung cancer, and prostate cancer after excluding the first 3 years of follow-up in the UK Biobank.

| Cancer site | Slow | Average | Brisk | *P*_trend_^a^ |
| --- | --- | --- | --- | --- |
| **Any cancer** |  |  |  |  |
| Model 1, HR (95% CI)^b^ | 1 (ref) | 0.87 (0.84, 0.90)** | 0.80 (0.78, 0.83)** | <0.001 |
| Model 2, HR (95% CI)^c^ | 1 (ref) | 0.93 (0.89, 0.96)** | 0.88 (0.85, 0.92)** | <0.001 |
| Model 3, HR (95% CI)^d^ | 1 (ref) | 0.93 (0.90, 0.97)** | 0.89 (0.85, 0.93)** | <0.001 |
| **Lung cancer** |  |  |  |  |
| Model 1, HR (95% CI)^b^ | 1 (ref) | 0.47 (0.42, 0.51)** | 0.31 (0.28, 0.35)** | <0.001 |
| Model 2, HR (95% CI)^c^ | 1 (ref) | 0.64 (0.58, 0.71)** | 0.49 (0.43, 0.56)** | <0.001 |
| Model 3, HR (95% CI)^d^ | 1 (ref) | 0.65 (0.58, 0.72)** | 0.50 (0.44, 0.57)** | <0.001 |
| **Prostate cancer in men** |  |  |  |  |
| Model 1, HR (95% CI)^b^ | 1 (ref) | 1.23 (1.12, 1.35)** | 1.29 (1.19, 1.40)** | <0.001 |
| Model 2, HR (95% CI)^c^ | 1 (ref) | 1.13 (1.03, 1.24)* | 1.15 (1.04, 1.27)** | 0.034 |
| Model 3, HR (95% CI)^d^ | 1 (ref) | 1.12 (1.02, 1.23)* | 1.14 (1.03, 1.25)* | 0.06 |

*CI*, confidence interval; *HKOS*, Hong Kong Osteoporosis Study; *HR*, hazard ratio. **p*<.05; ***p*<.01.

1. Adjusted for age and sex.
2. Model 1 + height, body mass index, baseline assessment center (UK Biobank only), ethnicity, education, deprivation (UK Biobank only), smoking, alcohol consumption, physical activity, and family history of cancer.
3. Model 2 + grip strength.

# **Supplementary Table 9.** Association between walking speed and risk of any caner after excluding the first 3 years of follow-up in the Hong Kong Osteoporosis Study.

| Any cancer | <1.0 m/s | ≥1.0 m/s |
| --- | --- | --- |
| Model 1, HR (95% CI)^a^ | 1 (ref) | 0.39 (0.18, 0.67)* |
| Model 2, HR (95% CI)^b^ | 1 (ref) | 0.35 (0.18, 0.67)* |
| Model 3, HR (95% CI)^c^ | 1 (ref) | 0.36 (0.19, 0.70)* |

*CI*, confidence interval; *HR*, hazard ratio; *SD*, standard deviation. **p*<.05.

1. Model 1: Adjusted for age and sex.
2. Model 2: Model 1 + height, body mass index, ethnicity, education, smoking, alcohol consumption, physical activity, and family history of cancer.
3. Model 3: Model 2 + grip strength.
